# Adaptation to Hot and Humid Climates in the Silkworm: Energy Reallocation and Cuticle Transpiration

**DOI:** 10.3390/insects16090962

**Published:** 2025-09-12

**Authors:** Jiajun Zhuo, Yuli Zhang, Xing Gao, Cailin Liang, Guizheng Zhang, Lihui Bi, Wei Wei, Shoumin Fang, Xiaoling Tong, Fangyin Dai, Cheng Lu, Quanyou Yu

**Affiliations:** 1 School of Life Sciences, Chongqing University, Chongqing 400044, China; 202326131046@stu.cqu.edu.cn (J.Z.); gx_3933@163.com (X.G.); liangcl123456@outlook.com (C.L.); 2 Guangxi Key Laboratory of Sericultural Genetic Improvement and Efficient Breeding, Nanning 530007, China; zyl8324@126.com (Y.Z.); zhangdoudou1999@163.com (G.Z.); gxblh@163.com (L.B.); gxcanyeweiwei@126.com (W.W.); 3 College of Life Science, China West Normal University, Nanchong 637002, China; fangshoumin@126.com; 4 State Key Laboratory of Silkworm Genome Biology, Institute of Sericulture and Systems Biology, Southwest University, Chongqing 400715, China; xltong@swu.edu.cn (X.T.); fydai@swu.edu.cn (F.D.); lucheng@swu.edu.cn (C.L.)

**Keywords:** tropical and subtropical humid climates, heat tolerance, *Bombyx mori*, transcriptomic response, adaptation

## Abstract

**Simple Summary:**

Global warming poses a significant threat to various organisms. The mechanisms of adaptation to heat environments, especially hot and humid climates, are poorly understood in insects. Plastic response plays important roles in responding to sudden high temperature damage. Genetic adaptation is crucial for sustained heat tolerance, but little is currently known about the specific genes involved, especially in tropical climates, where high temperatures and high humidity combine to create additional stress. This study explores the thermal genetic adaptation and plastic response mechanisms of silkworm resources that have long been adapted to high temperature and humidity regions. Our findings not only deepen our understanding of how insects adapt to global warming but also provide support for breeding heat-resistant silkworms.

**Abstract:**

The silkworm (*Bombyx mori*) is rich in germplasm resources, including thermotolerant strains that live in tropical/subtropical humid climates. In this study, two thermotolerant strains and one sensitive strain were used as materials, with the former exhibiting higher critical thermal maximum (CTmax) values. Under different temperature and humidity stresses, physiological and transcriptomic responses of the fifth instar larvae were compared. It was confirmed that high humidity exacerbates harmful effects only under high temperature conditions. Based on transcriptome and co-expression network analysis, 88 evolved thermoplastic genes (Evo_TPGs) and 1338 evolved non-plastic genes (Evo_non-PGs) were identified, which exhibited specific responses or expressions in the two thermotolerant strains. Eighteen of the Evo_TPGs encode cuticular proteins, 17 of which were specifically downregulated in the two thermotolerant strains after short-term exposure to 35 °C. This may promote cuticular transpiration to dissipate internal heat, thus compensating for the suppression of tracheal ventilation in hot and humid climates. For the Evo_non-PGs, most of the metabolic genes showed lower expression at background levels in the thermotolerant strains, while oxidative stress genes showed the opposite trend, suggesting that silkworms can enhance heat tolerance by suppressing metabolic rates and allocating more resources to overcome heat-induced oxidative damage. Furthermore, the heat resistance-related genes showed higher single nucleotide polymorphisms (SNPs) between resistant and sensitive strains compared to randomly selected genes, suggesting that they may have been subjected to natural selection. Through long-term adaptive evolution, thermotolerant silkworms may reduce their internal temperature by dynamically regulating cuticle respiration in response to high temperature and humidity, while allocating more energy to cope with and repair heat-induced damage. Overall, these findings provide insights into the evolution of heat-resistant adaptations to climate change in insects.

## 1. Introduction

Global warming has become a pressing global concern, with average temperatures rising by approximately 0.2 °C per decade compared to pre-industrial levels (1850–1900). Climate projections suggest that if this trend continues, global temperatures could reach 1.5 °C by 2040 and 2.0 °C by 2100. The impacts of climate warming have been found to have profound consequences, affecting species distributions, reproductive patterns, community composition, and ecosystem functions on a global scale [1,2] and are likely to alter fundamental species interactions, leading to cascading effects on species abundance [3]. Additionally, mortality and biodiversity loss resulting from global warming may further exacerbate climate change [4].

Although insects represent the most diverse group of animals, studies have revealed that approximately 40% of the world’s insect species are at risk of extinction in the coming decades due to multiple factors, including global warming [5]. However, it should be noted that these insects are not passive recipients of environmental changes. For instance, grasshoppers can endure air temperatures that exceed their lethal internal temperatures for over 1 h, primarily due to cuticular transpiration and the ventilation mechanisms of their tracheal system, which help maintain their internal body temperature below that of the surrounding air [6]. In addition, insects exhibit plastic responses to short-term high temperature exposure that can enhance their survival and increase their tolerance to future heat stress through acclimation [4,7,8].

Plastic response alone may not be sufficient to cope with the ongoing impacts of global warming [9,10,11]. Frequent extreme high temperatures can drive the evolution of thermal tolerance, enabling populations to buffer the effects of heat [12] and reduce the risk of extinction [10]. Therefore, genetic adaptation, resulting from both new mutations and existing genetic variation, is essential for developing thermal resistance [13,14,15]. In this regard, Takahashi et al. identified 19 quantitative trait loci associated with heat resistance in a tolerant strain of *Drosophila melanogaster* [16]. Similarly, Waldvogel et al. used population genetic analysis to investigate positive selection in the genomes of heat-resistant and sensitive populations of *Chironomus riparius*, identifying 162 candidate heat-resistant genes [14]. Despite these insights, research on heat-resistant loci or genes remains limited, highlighting the need for further investigation into the underlying mechanisms.

In addition to temperature, ambient humidity may affect animal adaptation and geographic distribution [17,18]. Tropical and subtropical regions are particularly characterized by high temperatures and elevated humidity levels [19,20]. Related studies have shown that humans can effectively dissipate heat through evaporative cooling even at high air temperatures and low moisture levels [21]. However, in hot and humid conditions, the effectiveness of evaporative cooling decreases, making it challenging to maintain a stable core temperature. Thus, high humidity adversely impacts thermoregulation in organisms and exacerbates negative effects on production and animal welfare [6,19,22]. Recent studies have started to explore the stress responses of insects under high temperature and humidity conditions [23,24,25]. For instance, transcriptome analysis in *Apis mellifera* identified 266 differentially expressed genes (DEGs) enriched in biosynthesis, metabolism regulation, and gated channel activity [24]. Additionally, temperature-activated transient receptor potential (TRP) ion channels have been shown to respond to various stimuli and may serve as key integrators of sensory information related to environmental temperature and humidity [26]. Therefore, similar to heat-tolerant genes, the presence of humidity-tolerant genes in organisms adapted to tropical humid climates deserves further investigation.

Adaptation is crucial for organisms living in extreme climatic conditions, as it typically requires numerous generations and sustained persistence [27]. Despite these challenges, some species and populations thrive in tropical and subtropical regions, having evolved thermal resistance. In the context of global warming, numerous studies have explored the plastic response mechanisms of individual populations or strains under high temperature conditions. However, understanding of heat-resistant genes and their underlying mechanisms remains limited, particularly regarding evolutionary adaptations to tropical and subtropical climates.

*Bombyx mori*, a domesticated silkworm, represents an important model organism with a rich diversity of local germplasm, including tropical polyvoltine strain [28]. Some researchers have analyzed the transcriptome of silkworms after high temperature stimulation, identifying a large number of heat shock proteins, antioxidant enzymes, detoxification enzymes, and other encoded genes that exhibit stress response expression [23,29,30]. However, in addition to heat stress response genes, do thermotolerant silkworms possess heat-resistant genes? In addition, thermotolerant silkworms are often exposed to high temperatures, and their mechanisms for dissipating heat and reducing heat damage are worth further exploration. In this study, we utilized two resistant strains derived from local polymorphic germplasm in tropical humid regions, with the thermal-sensitive strain Haoyue (HY) as an experimental model to elucidate the evolutionary responses and molecular networks that facilitate adaptation to hot environments in silkworms. Our findings may facilitate molecular breeding efforts in silkworms and deepen the understanding of evolutionary adaptation to tropical climates in insects.

## 2. Materials and Methods

### 2.1. Insect Rearing

The resistant strains 7532 and 932 were derived from local polymorphic germplasm adapted to tropical and subtropical humid regions of China. The HY strain, which is commonly used for silk production in temperate climates, is highly sensitive to thermal stimulation. Eggs from all three strains were obtained from the Guangxi Sericulture Technology Extension General Station of China. Under standard rearing conditions, silkworms were housed at 25 ± 1 °C with 70% ± 3% relative humidity (RH) and a photoperiod of 14 h light and 10 h dark. All silkworms were fed fresh mulberry leaves.

### 2.2. Exposure of Silkworms to Different Temperatures and Humidities

The male and female individuals of each strain were separated on day 0 of the fifth instar, with female individuals having four transparent spots on the ventral surface of the eighth and ninth abdominal segments. To assess survival probability throughout the entire fifth instar, the male and female larvae of each strain were subjected to the same treatment; namely, they were reared under 35 ± 0.5 °C with 70 ± 3% RH and 35 ± 0.5 °C with 90 ± 3% RH, respectively. Mortality was monitored for 7 days, and the number of dead insects was counted daily. To simulate the effects of temperature and humidity on yield traits, moderate-high temperature (30 °C) and high humidity (90% RH) were selected to expose the fifth instar larvae. Three replicates were set for both male and female individuals, with 30 individuals in each replicate.

### 2.3. Measuring CTmax

Critical thermal maximum (CTmax) is a crucial parameter for assessing heat tolerance in insects, including ants, bed bugs, and spiders [31,32,33]. Laboratory trials for determining the critical thermal maximum (CTmax) were conducted using a Thermal-Lok 2-position Dry Heat Bath (USA Scientific #2510-1101, New York, NY, USA). Twenty male and twenty female individuals on day 2 of the fifth instar (5L2D) under normal rearing conditions of 25 °C and 70% RH for each strain were tested. Each individual was placed in a 15 mL Eppendorf tube, the bottom of which was filled with modeling clay, and then loaded into the dry bath. An empty tube was designated as a control chamber to monitor the temperature in the remaining chamber. The temperature in the control tube was recorded using a single-channel thermocouple data logger (HOBO UX100-014M, Onset Computer Corporation, Bourne, MA, USA). Silkworms were acclimated in the chambers at a starting temperature of 30 °C for 5 min. The dry bath was then heated at a constant rate of 1 °C every 5 min until the upper thermal limits for all trial individuals were determined. The CTmax was defined as the temperature at which each individual lost muscle contraction and ceased body movement.

### 2.4. Histological Analysis

Male individuals at day 2 of the fifth instar (5L2D) were exposed to treatments of 35 °C/70% RH and 25 °C/70% RH for 24 h. After treatment, the midguts of the individuals were dissected and fixed in 4% paraformaldehyde (PFA) for 12 h. The fixed midguts were then embedded in paraffin blocks, and sections were cut at a thickness of 7–10 μm using a Leica RM2135 microtome (Leica, Deer Park, IL, USA), and the midgut sections were stained with hematoxylin and eosin (H&E) and subsequently viewed and photographed using a digital camera mounted on an Olympus Microscope (Olympus EX51, Tokyo, Japan).

### 2.5. RNA-Sequencing and Analyzing DEGs

To investigate the molecular basis of adaptive evolution in high temperature and high humidity environments, 30 male larvae from each strain were exposed for 24 h under 25 °C/70% RH, 25 °C/80% RH, 25 °C/90% RH, 30 °C/70% RH, 35 °C/70% RH and 35 °C/90% RH, respectively. The whole bodies were collected after removing the digestive tract. Each individual was stored separately in RNAlater (Ambion, Austin, TX, USA) and stored at −80 °C. Total RNA was extracted with TRIzol reagent (Invitrogen, Carlsbad, CA, USA). RNA quality was checked by an Agilent Bioanalyzer 2100 (Agilent, Santa Clara, CA, USA). Each RNA sample was composed of three individuals with equal amounts of RNA. Two biological replicates were set for RNA samples of resistant strains, while three biological replicates were set for the sensitive strain. The construction of a standard Illumina sequencing library and paired-end sequencing was performed by Beijing Novogene Technology Co., Ltd., Beijing, China.

The raw reads were filtered using NGS QC Toolkit (https://github.com/mjain-lab/NGSQCToolkit, accessed on 20 January 2022), including reads with more than 30% low quality bases (Q < 30) and reads with more than 10% N. Clean reads were aligned to the reference genome of the silkworm [34] using HISAT 2.2.0 with default parameters. Read counts for each sample were generated by HTSeq v 2.0.1. Gene expression levels were estimated using FPKM values (fragments per kilobase of exon per million fragments mapped). Differentially expressed genes (DEGs) were identified using the edgeR version 3.14.0 with thresholds: |log2(fold change)| ≥ 1 and Benjamini-Hochberg adjusted *p*-value < 0.05.

### 2.6. Functional Annotation

Open reading frames (ORFs) of the transcripts were predicted using TransDecoder v5.5.0. The predicted protein sequences were then subjected to homologous searches against PFAM protein sequences using the hmmscan tool in HMMER v3.2.1 software. For functional annotation, KEGG orthology (KO) assignments and pathway reconstruction were performed using the BlastKOALA server (https://www.kegg.jp/blastkoala/, accessed on 25 October 2023). The protein pathways identified in this analysis were annotated through BlastKOALA. Additionally, Gene Ontology (GO) enrichment analysis was conducted using the STRING v12.0 database (https://cn.string-db.org/, accessed on 26 October 2023), while KEGG enrichment analysis was carried out using KOBAS 3.0 (http://kobas.cbi.pku.edu.cn/, accessed on 26 October 2023).

### 2.7. Gene Co-Expression Network Analysis

Weighted Gene Correlation Network Analysis (WGCNA) was conducted to identify significant clusters and module eigengenes with similar expression patterns [35]. In this study, we utilized 18 samples and 5655 DEGs (Table S2) to construct a co-expression network using the WGCNA v1.63 package in R. The weighted adjacency matrix was transformed into a topological overlap metric matrix (TOM) to estimate connectivity within the network. The minimum gene module size was set to 25 to obtain appropriate modules, using a deepSplit threshold of 2. A soft threshold of 16 was applied, while other parameters were maintained at their default settings. The association of modules with traits was assessed by calculating gene significance (GS) and module membership (MM). Visualization of the gene co-expression regulatory networks within target modules was performed using Cytoscape v3.10.0 software.

### 2.8. Mfuzz Clustering

The average FPKM values of the replicates were used as expression data to perform expression pattern clustering. FPKM values for each sample were combined into a single expression matrix. Subsequently, soft clustering across samples was executed using the Mfuzz v2.60.0 R package. The optimal number of clusters was determined using the minimum centroid distance method within the Mfuzz package.

### 2.9. Transcriptional Regulatory Network Analysis

The 2 kb upstream regions of the target gene set were retrieved from the reference genome of the silkworm [34] using BEDTools [36]. Enrichment of transcription factor (TF) binding motifs in the cis-regulatory sequences was analyzed using CentriMo with insect motifs from JASPAR CORE (2022) available on the MEME Suite server (https://meme-suite.org). The enriched motifs were subsequently scanned in the upstream regions of the target gene set using FIMO, with an e-value threshold set at <1 × 10^−5^ on the MEME Suite server. Potential TFs were predicted using the AnimalTFDB database (http://bioinfo.life.hust.edu.cn/AnimalTFDB4/, accessed on 10 November 2023). The relationships between the enriched motifs and TFs were manually verified in the JASPAR database (https://jaspar.genereg.net/, accessed on 10 November 2023). Potential regulatory relationships between each TF and the target genes were established. The functional categories of the TFs’ target genes were revealed using BlastKOALA. Finally, the regulatory network of TFs was visualized using Cytoscape v3.10.0.

### 2.10. Tissue Enrichment Analysis

The transcriptome datasets of different tissues of the Dazao strain on day 3 of the fifth instar larvae have been released (Table S1; https://silkdb.bioinfotoolkits.net/, accessed on 30 November 2023). We downloaded the raw reads of the RNA-seq data from NCBI SRA (https://www.ncbi.nlm.nih.gov/sra, accessed on 30 November 2023). After filtration, clean reads were mapped to the silkworm reference genome using HISAT 2.2.0. FPKM values were calculated for each gene assembled from the present RNA-seq datasets. Tissue enrichment analysis refers to Hsu et al., 2020 [37]. Briefly, the highly expressed genes were required to be at least twice as high as the average level in all tissues. Taking evolutionary thermoplastic genes as a target gene set, the number of highly expressed thermoplastic genes divided by the number of non-highly expressed genes was used to calculate the odds value. DEGs unrelated to any of the target genes were used as comparison. Fisher’s exact test was applied for the enrichment of tissue expression.

### 2.11. Analyzing Nucleotide Divergence of Genes and Cis-Regulatory Regions

Genomic DNA of the three strains’ pupae was extracted using a DNeasy Blood and Tissue Kit (Qiagen, Manchester, UK). Construction of a standard Illumina sequencing library and paired-end sequencing were performed by Beijing Novogene Technology Co., Ltd. Approximately 15× coverage for each sequenced sample was obtained. The raw reads were filtered using NGS QC Toolkit, and clean reads were aligned to the silkworm reference genome [34] using BWA-MEM version 0.7.13 with default parameters [38]. SNP calling was performed according to the recommendations of the Genome Analysis Toolkit (GATK) v3.6.1 [39]. Briefly, two processes were conducted before SNP calling, including sorting mapped reads using SAMtools [40] and removing PCR duplicates with Picard (https://broadinstitute.github.io/picard/, accessed on 30 November 2023). Then, SNP variants were called by the UnifiedGenotyper module variant call format (VCF) and used for further analysis. In GATK v3.6.1. SNPs were filtered with the following cutoffs: ReadPosRankSum < 4, MQRankSum < 5, BaseQualityRankSum < 3, ReadPosRankSum < 4, and FS > 50. SnpEff software is an annotation tool for genomic variants, which annotates variants based on their genomic locations and predicts coding effects [41]. The effect of each variant site from the VCF file was annotated using SnpEff v4.3t. We focused on the variants in the gene and its 2 kb upstream (cis-regulatory) region.

### 2.12. Statistical Analysis

Statistics were performed using the SPSS 18.0 software package (SPSS Inc., Chicago, IL, USA) unless otherwise stated. Statistical analyses were performed by one-way analysis of variance (ANOVA) to assess significance, including comparisons of yield traits and lethality rates. Differences were considered to be significant at *p* < 0.05. Multiple comparisons were detected by the post hoc test with Bonferroni corrections under *p* < 0.05, such as nucleotide divergence. In addition, survival probability was compared using the log-rank test in R v4.1.3. Principal component analysis (PCA) was also conducted in R v4.1.3. The ggplot function in R to analyze the relationship between CTmax and weight, in which a linear regression model was used.

## 3. Results

### 3.1. Thermal Tolerance Assessment and Physiological Experiments

**Figure 1:**
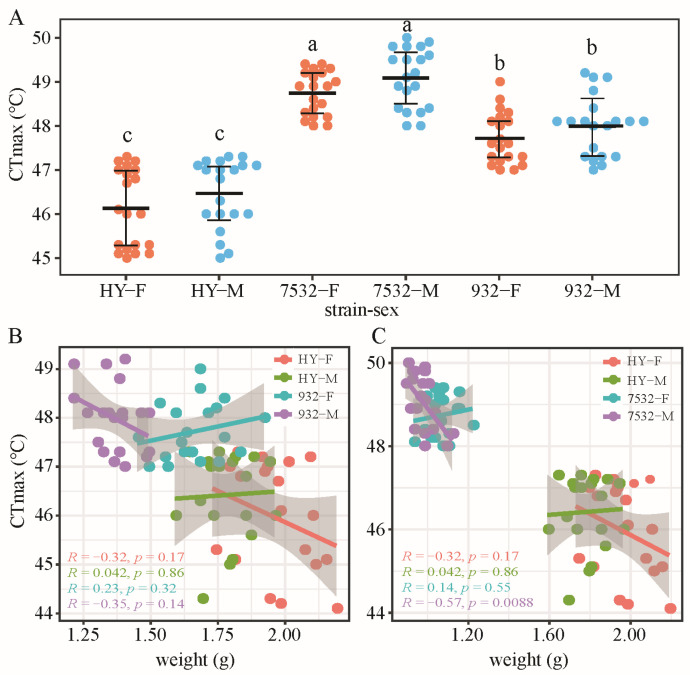
Comparison of CTmax between the resistant and sensitive strains. (**A**) Beeswarm plot of CTmax on day 2 of the fifth instar larvae. The bolded horizontal lines represent mean values. The error bars represent standard deviation. HY: Haoyue. F and M after the strain name represent females and males, respectively. Multiple comparisons were detected by the post hoc test with Bonferroni corrections. The a, b and c indicate significant differences at *p* < 0.05. (**B**) Relationship between CTmax and individual weight in the HY and 932 strains. Linear regression and a 95% confidence interval are shown. (**C**) Relationship between CTmax and weight of HY and 7532.

The results indicated that the average CTmax values for the two thermotolerant strains ranged from 47.72 ± 0.59 °C to 49.09 ± 0.67 °C (Figure 1A), significantly higher than that of the sensitive strain HY. We also analyzed the relationship between CTmax and individual weight (Figure 1B,C), finding no significant correlation, except in the males of strain 7532.

**Figure 2:**
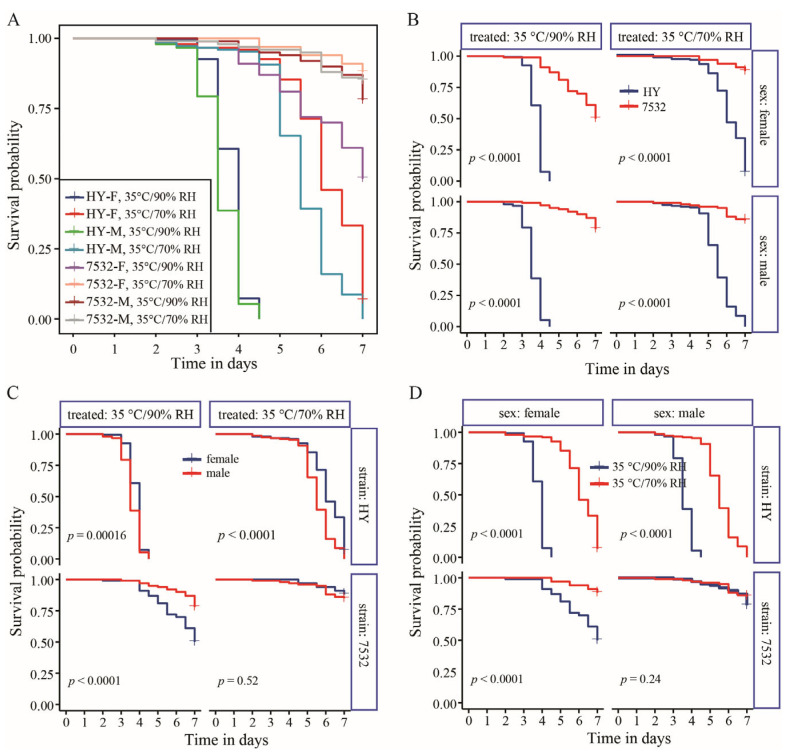
Survival probability between the resistant strain 7532 and sensitive strain Haoyue under high temperature and humidity treatment. (**A**) Summary of all the survival curves. The curves were fitted using the Kaplan–Meier method of the Survfit function in the Survival package. The *x*-axis represents the number of treatment days for the fifth instar larvae. (**B**) Differences in survival probability between 7532 and Haoyue (HY). The *p*-values were computed using the log-rank test. (**C**) Comparison of the survival probability between males and females. (**D**) Survival probability under two treated conditions.

**Figure 3:**
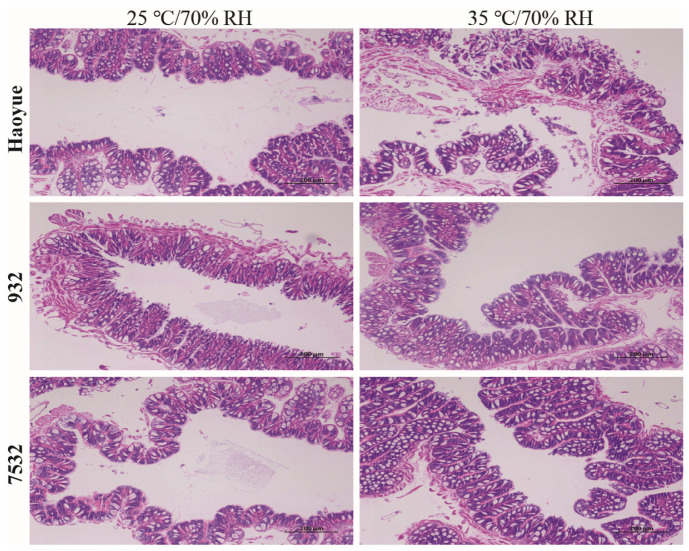
The H&E stain of the midgut sections after high temperature treatment. The midguts were isolated from the male individuals on day 3 of the fifth instar larvae after 24 h treated at 35 °C/70% RH and normal conditions (25 °C/70% RH).

It was indicated that the thermotolerant strains showed higher survival probabilities than those of the sensitive strain regardless of the treatment conditions or sex (Figure 2B and Figure S1B). Under high temperature conditions, high humidity significantly increased mortality rates for both thermotolerant and sensitive strains, except for males of the 7532 strain (Figure 2D and Figure S1D). However, comparing the mortality rates at 25 °C/90% RH with those at 25 °C/70% RH revealed that the higher humidity (90% RH) did not lead to a significant increase in mortality. After acute heat stress at 35 °C and 70% RH for 24 h, midgut sections stained with H&E showed significant cell sloughing into the lumen of the sensitive HY larvae (Figure 3). A similar occurrence has been reported in the midgut of honey bees subjected to 45 °C for 4 h [42]. In contrast, no sloughing of midgut cells was observed in the two resistant strains.

**Figure 4:**
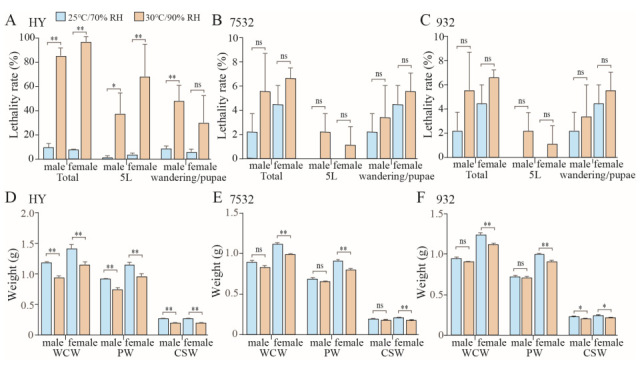
Effects of moderate-high temperature and high humidity on yield traits and lethality rate. (**A**–**C**) Comparison of lethality in the silkworms after moderate-high temperature and high humidity. The fifth instar larvae (5L) were kept at 30 °C/90% RH and then kept under 25 °C/70% RH during the wandering and pupal stages. Lethality rates were counted at 5L and wandering/pupal stages, respectively. (**D**−**F**) Effects on cocoon and silk production in Haoyue (HY), 7532 and 932. Whole cocoon weight (WCW), pupal weight (PW), and cocoon shell weight (CSW) were investigated on day 5 of the pupal stage. The graph shows the average yield data for each individual. * *p* < 0.05; ** *p* < 0.01; ns: not significant.

Almost all individuals of the sensitive strain Haoyue will die after 7 days of exposure to a 35 °C high temperature (Figure 2C). To simulate the effects of temperature and humidity on yield traits, moderate-high temperature (30 °C) and high humidity (90% RH) were selected to expose the fifth instar larvae, ensuring that there were enough surviving individuals for data statistics. In general, the total lethality rate of sensitive strain Haoyue exceeded 80%, while those of resistant strains were less than 10% (Figure 4A–C). Notably, the growth of HY was severely inhibited, resulting in a nearly 20% reduction in pupal weight (PW) (Figure 4D). The yield traits, including whole cocoon weight (WCW) and cocoon shell weight (CSW), were also significantly decreased, with CSW reduced by nearly 28% (Figure 4D). For the resistant strains, PW, WCW, and CSW significantly decreased only in female individuals (Figure 4E,F), with the reduction being relatively minor; for example, strain 7532 exhibited reductions of 11.98%, 12.25%, and 13.46%, respectively. Furthermore, significant decreases in spawning numbers and hatchability were observed only in the sensitive strain (Figure S3).

### 3.2. Overview of Transcriptome Experiment

**Figure 5:**
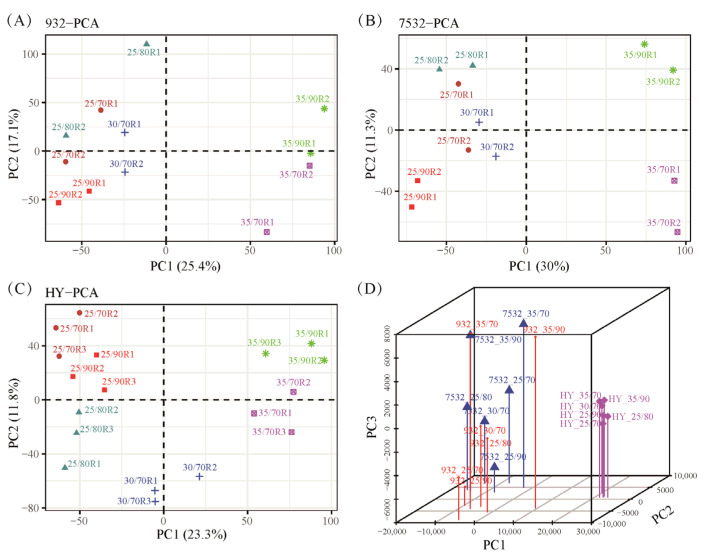
Principal component analysis of all the transcriptomic samples. (**A**–**C**) PCA analysis of the 932, 7532, and Haoyue. R1, R2, and R3 represent the sample replicates. The 25/70, 25/80, 25/90, 30/70, 35/70, and 35/90 represent the treatment conditions 25 °C/70% RH, 25 °C/80% RH, 25 °C/90% RH, 30 °C/70% RH, 35 °C/70% RH, and 35 °C/90% RH. (**D**) Three-dimensional (3D) principal component analysis. The average FPKM values of each sample were used for 3D plotting with the scatterplot3d package in R v4.1.3.

RNA sequencing of the whole bodies was conducted, and principal component analysis (PCA) demonstrated that sample replicates were closely clustered (Figure 5). The treatments at 35 °C/70% RH and 35 °C/90% RH were distinctly separated from the other treatments (Figure 5A–C). Additionally, samples from the resistant strains were clearly distinguished from those of the sensitive strain (Figure 5D). In total, 16,449 assembled genes were expressed in at least one sample. By comparing different treatments within each strain, differentially expressed genes were identified. A total of 5655 DEGs were found across the three strains after exposure to varying temperatures and humidity levels (Table S2).

### 3.3. Specific Thermoplastic Genes of the Resistant Silkworms Adapted to a High Temperature Environment

**Figure 6:**
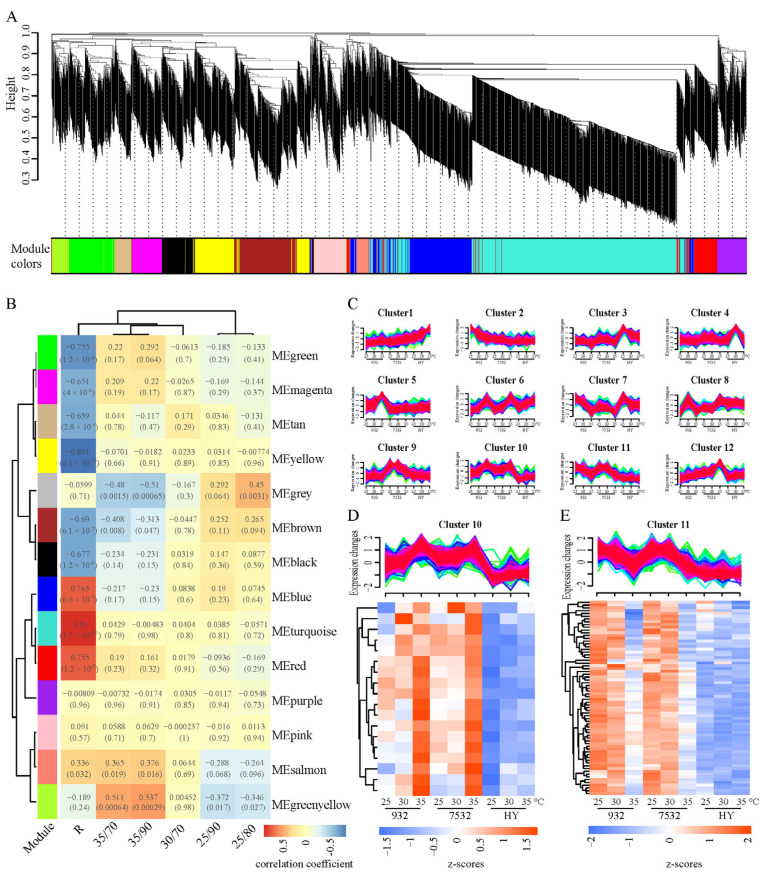
Co-expression analysis for differentially expressed genes after temperature and humidity exposure. (**A**) Cluster dendrogram of the co-expression network modules. Each vertical line represents a single gene. The *y*-axis represents the value of the distance measurement between clusters. The module below corresponds to the height above, i.e., different heights correspond to different modules. (**B**) Correlation between modules and traits according to Pearson correlation. R represents the thermotolerance (resistance) trait. The *p*-values of Pearson correlation are shown in parentheses. (**C**) Soft clusters of all the DEGs respond to heat stress by Mfuzz software. Red and purple lines correspond to genes with high membership values. The numbers on the *x*-axis represent the treated temperatures, including 25 °C, 30 °C, and 35 °C. (**D**) Heatmap of the overlapping genes between cluster 10 and the significant related modules (MEblue, MEturquoise, and MEred) of the R trait. (**E**) Heatmap of the overlapping genes between cluster 11 and the significantly related modules of the R trait.

In this study, all 5655 thermal and humidity response genes were included in the WGCNA analysis (Figure 6A), and the results revealed that the turquoise, blue, and red modules exhibited the strongest correlations with the resistant strains, leading to the identification of 1025 plastic genes (Figure 6B). Furthermore, all thermoplastic response genes from the three strains were subjected to soft clustering using the Mfuzz package (Figure 6C). Clusters 10 and 11 displayed similar expression patterns between the two resistant strains, which differed from those of the sensitive strain. The genes in Clusters 10 and 11 were cross-referenced with the resistance-related modules identified in the WGCNA, resulting in the identification of 18 upregulated and 70 downregulated genes, respectively (Figure 6D,E). Thus, a total of 88 genes specific to thermal stress response in resistant silkworms were identified, suggesting they may have evolved during adaptation to high temperature environments; these genes were designated as evolved thermoplastic genes (Evo_TPG) (Table S3).

KEGG enrichment analysis indicated that Evo_TPG genes were primarily enriched in pathways related to sugar metabolism, immunity, and proteolysis, including starch and sucrose metabolism, glycolysis/gluconeogenesis, phagosome, and ubiquitin-mediated proteolysis (Table S4). GO enrichment analysis revealed significant associations with the pyruvate metabolic process, the carbohydrate catabolic process, and the generation of precursor metabolites and energy (Table S5). Notably, 18 of the 88 Evo_TPG genes were identified as cuticular proteins (Table S3), with high expression levels in the head and epidermis of the 5L3D larvae (Figure S4). Importantly, 17 of these cuticular proteins were specifically downregulated in the resistant silkworms under high temperature stress, suggesting a potential decrease in cuticle thickness. Overall, these findings indicate that resistant strains have evolved specific genes that respond to high temperatures, which may play a crucial role in enhancing survival.

### 3.4. Pervasive Thermal Response Mechanisms Shared by Resistant and Sensitive Silkworms

In addition to the genes specifically responding to thermal stimuli in the resistant strains, we identified clusters 1, 2, 3, 6, and 7 in Figure 6C, which displayed similar stress response patterns in both the resistant and sensitive strains. A total of 146 thermoplastic genes (82 downregulated and 64 upregulated) were identified. They demonstrated identical stress responses in both resistant and sensitive strains and were designated as TPG_RS (Table S3; Figure S5A). KEGG enrichment analysis revealed significant over-representation in eight pathways, including protein processing in the endoplasmic reticulum, ribosome biogenesis in eukaryotes, pyrimidine metabolism, and longevity regulation (Table S4; Figure S5B). GO enrichment analysis indicated that the enriched categories included the Box C/D RNP complex, sno(s)RNA-containing ribonucleoprotein complex, preribosome, and drug metabolism (Table S5; Figure S5B). Previous studies have established that HSPs are among the most common genes involved in heat stress response [4]. In this study, despite utilizing three silkworm strains with different genetic backgrounds, we identified six HSP genes that exhibited consistent expression patterns in response to heat stress (Table S3). This finding further supports the notion that heat shock proteins play a general role in responding to thermal stimuli, particularly in protecting proteins from damage.

### 3.5. Evolved Humid-Plastic Genes in the Resistant Silkworms

To investigate the potential mechanisms of silkworm adaptation to high humidity, we analyzed the 969 DEGs that were subjected to varying humidity levels at 25 °C using the Mfuzz package for clustering (Figure S6). However, we were unable to identify consistent expression trends for the two resistant strains (Figure S6). Consequently, we utilized Venn diagrams to analyze the DEGs across each strain (Figure S7A). This analysis revealed that six DEGs were shared among all three strains, but they did not exhibit consistent expression patterns (Figure S7B). Additionally, 36 DEGs were shared between the two resistant strains, of which 10 displayed similar expression patterns that differed from those of the sensitive strain (Figure S7C). These 10 genes are potential candidates for specific responses that have evolved as a result of long-term adaptation in resistant silkworms and have been designated as evolved humid-plastic genes (Evo_HPG).

### 3.6. Evolved Non-Plastic Genes (Evo_non-PG) Improve Thermal Tolerance in Resistant Silkworms

**Figure 7:**
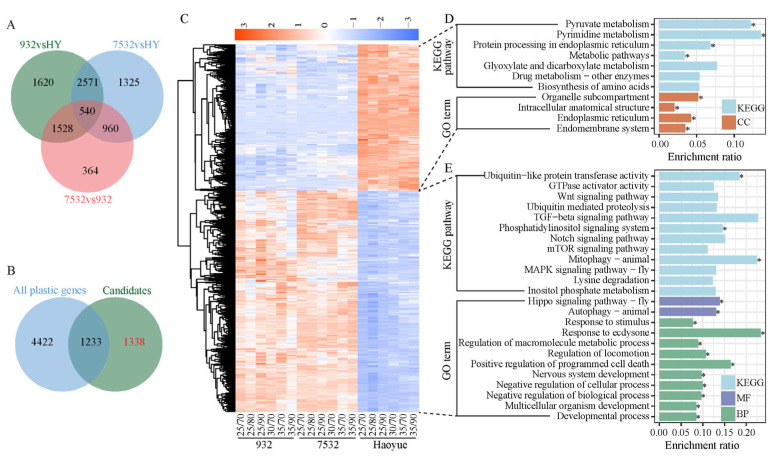
Identification and expression heatmap of the Evo_non-PG genes. (**A**) Venn diagram of the DEGs among the three strains under normal rearing conditions. In total, 2571 genes were the overlapping DEGs between the resistant and sensitive strains, which were the candidates for Evo_non-PG genes. HY: Haoyue. (**B**) Venn diagram between the candidates of Evo_non-PG genes and all the 5655 plastic response genes. (**C**) Expression heatmap of the 1338 Evo_non-PG genes. (**D**,**E**) Representatives of the enriched KEGG pathways and GO terms for the downregulated and upregulated Evo_non-PG genes in the resistant strains. CC: cellular component, BP: biological process, MF: molecular function. * FDR < 0.05.

**Figure 8:**
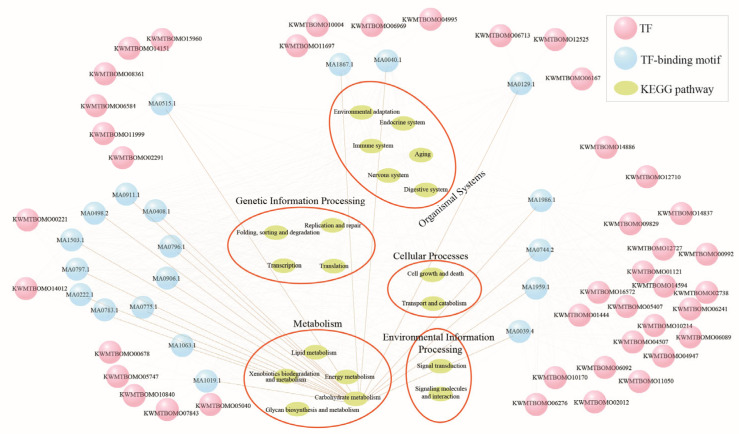
The predicted transcriptional regulatory network of the evolved non-plastic genes. The pink circles are transcription factors (TFs) of the Evo_non-PG genes. The blue circles represent the TF-binding motifs enriched at 2 kb upstream of all the protein-coding genes of the Evo_non-PG. The target genes of TFs were annotated to corresponding KEGG pathways using BlastKOALA (https://www.kegg.jp/blastkoala/, accessed on 25 October 2023).

In addition to specific stress-responsive gene modules, changes in basal gene expression represent an essential survival strategy for enhancing thermal tolerance. We compared DEGs among the three strains in normal rearing conditions (25 °C/70% RH) (Figure 7A). A total of 2571 common DEGs were identified between the resistant and sensitive strains, excluding any basal expression differences between the two resistant strains (Figure 7A). After further excluding heat and/or humidity plastic response genes (Figure 7B), we identified 1338 genes that represent the Evo_non-PG, which may mainly contribute to the heat tolerance of the resistant silkworms. The Evo_non-PG genes included 532 downregulated and 806 upregulated genes at the basal expression level in the resistant silkworms compared to the sensitive strain (Figure 7C; Table S3). The downregulated genes were primarily enriched in KEGG pathways related to protein translation and metabolism (Figure 7D; Table S4). Comparatively, the upregulated genes were mainly associated with KEGG pathways such as mTOR signaling, ubiquitin-mediated proteolysis, and growth-related signaling (Figure 7E; Table S4). A total of 67 TFs were identified within the Evo_non-PG genes, including 22 zinc-finger C2H2 (zf-C2H2), 8 BTB, and 5 basic helix-loop-helix (HLH) factors (Table S6). Notably, 20 TF-binding motifs were enriched in the 5′-flanking regulatory regions of the protein-coding genes of the Evo_non-PG, with nearly all of them containing at least one enriched motif (Figure 8).

### 3.7. Tissue Enrichment Expression of the Evolved and Thermoplastic Genes

**Figure 9:**
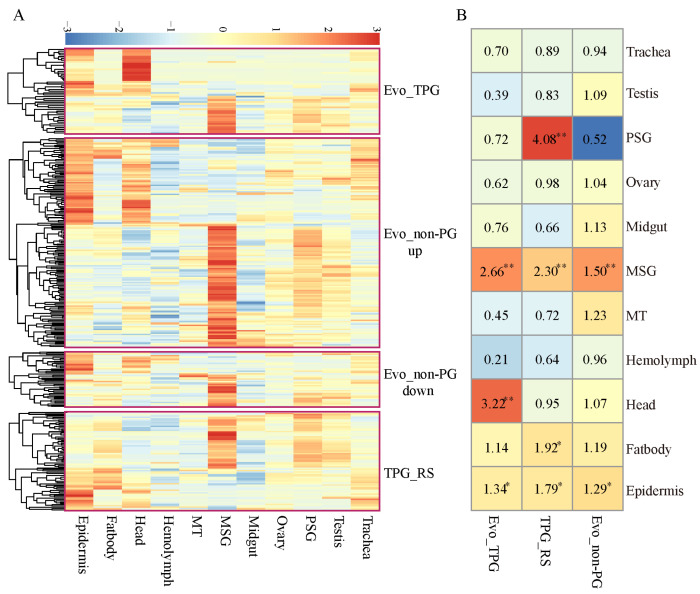
Tissue enrichment of the evolved and plastic response genes. (**A**) Heatmap of the expression levels for the tissue-enriched genes in various tissues on day 3 of the fifth instar larvae. MT: Malpighian tubule, MSG: middle silk gland, PSG: posterior silk gland. (**B**) Tissue enrichment of the evolved and plastic response genes. The colors and numbers denote the magnitude of the odds ratio. Each cell represents the result of a Fisher’s exact test. ** *p* < 0.01, * *p* < 0.05.

In this study, the entire body of the silkworms was used as tissue samples for RNA sequencing to gain a comprehensive understanding of the evolutionary responses to thermal and humid climates. Tissue enrichment analysis was conducted for the evolved and plastic genes in fifth instar larvae (5L3D), revealing the primary body regions where these genes exert their functions. A total of 47 genes from the Evo_TPG set, 204 from the Evo_non-PG set, and 75 from the TPG_RS set were highly expressed in at least one tissue (more than twice the average expression across all tissues) (Figure 9A). The highly expressed genes in the Evo_TPG set were predominantly enriched in the head, MSG, and epidermis (Figure 9B). Similarly, TPG_RS genes were enriched in the PSG, MSG, fat body, and epidermis. Interestingly, MSG and epidermis were common enriched tissues for Evo_non-PG, Evo_TPG, and TPG_RS genes, suggesting that these two tissues may play significant roles in thermal adaptation and acclimatization.

### 3.8. Nucleotide Divergence of the Evolved and Thermoplastic Genes

**Figure 10:**
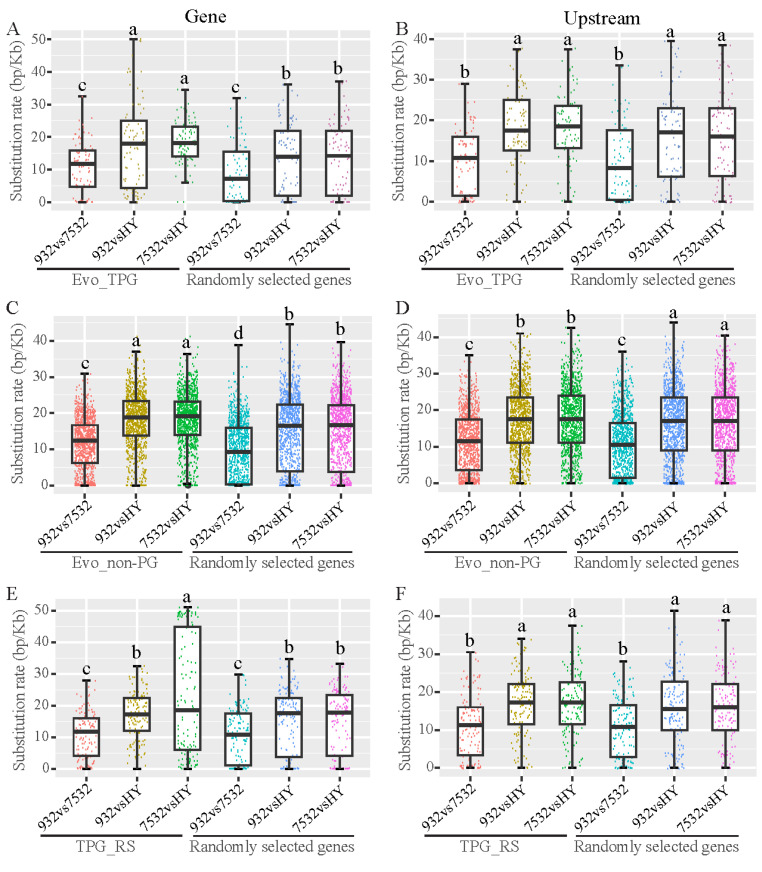
Nucleotide divergence of the evolved and plastic genes between the resistant and sensitive strains. HY: Haoyue. (**A**,**B**) Substitution rates of the gene region and 2 kb upstream of the Evo_TPG. Eighty-eight randomly selected genes were used for comparison. (**C**,**D**) Substitution rates of the gene region and 2 kb upstream of the Evo_non-PG. In total, 1338 randomly selected genes were used for comparison. (**E**,**F**) Substitution rates of the gene region and 2-kb. Multiple comparisons were detected by the post hoc test with Bonferroni corrections. The a, b and c indicate significant differences at *p* < 0.05.

Using the re-sequenced genome sequences of the three silkworm strains, we investigated the nucleotide substitution rates of Evo_TPG, Evo_non-PG, and TPG_RS. We compared SNPs in the genes and their upstream regions within the target gene sets among the three strains, utilizing a comparable number of randomly selected genes as controls (Figure 10). The substitution rate for Evo_TPG gene regions was found to be higher between the resistant and sensitive strains compared to the randomly selected genes, but this difference was not significant in the upstream regions (Figure 10A,B). In contrast, Evo_non-PG genes were found to have significantly greater nucleotide differences in both gene regions and upstream regulatory regions compared to the randomly selected genes between the resistant and sensitive strains (Figure 10C,D). However, the TPG_RS shared by the resistant and sensitive strains (TPG_RS) did not show a higher mutation rate, except for the gene region between strains 7532 and HY (Figure 10E,F).

## 4. Discussion

Temperature and humidity are important environmental factors influencing silkworm growth. In this study, we found that high temperature is a more detrimental environmental factor, with its impact exacerbated by elevated humidity levels, indicating that temperature is a key environmental factor for most ectothermic insects [4]. Previous studies have demonstrated that organisms tend to grow faster but reach smaller final body sizes at higher temperatures compared to lower temperatures [43]. This phenomenon may be attributed to the increased energy costs associated with coping with elevated temperatures. Similarly, our findings show that resistant strains of silkworms have lower larval and pupal weights compared to sensitive strains (Figure S9A–D). Moreover, traits related to cocoon yield, such as WCW and CSW, along with fecundity, were also significantly reduced in the resistant silkworms (Figure S9E–I). These results suggest a trade-off between thermal resistance and individual weight, cocoon yield, and fecundity traits. However, few studies have explored the trade-offs associated with thermal resistance, indicating that this area merits further investigation. In this study, the thermal-resistant evolutionary genes were categorized into two groups: (i) Evo_TPG, which exhibit concordant thermoplastic responses in resistant strains, and (ii) Evo_non-PG, which displays differential expression at the basal level between resistant and sensitive strains under normal rearing conditions. These evolutionary genes provide valuable insights into the mechanisms of thermal resistance and the associated trade-offs.

Recent studies on *Hydra* have shown that temperature-induced changes in cell number are regulated by Wnt signaling and TGF-β signaling, with cell number being the strongest determinant of body size [44]. In this present study, Wnt signaling was found to be the most significantly enriched pathway among the upregulated Evo_non-PG genes in the resistant strains (Table S4). The TGF-β signaling pathway was also enriched, comprising five upregulated genes. Notably, we identified an upregulated TF (*Sox*; *KWMTBOMO02291*) within the Evo_non-PG genes, which may regulate classical Wnt signaling [45,46]. The regulatory relationship between the Sox and Wnt signaling-related genes was examined (Figure 8), revealing that *Sox* interactions may precisely control the spatial and temporal expression of classical Wnt signaling [45]. It was suggested that these differentially expressed TFs may play an important regulatory role in heat tolerance. The smaller body size observed in the resistant silkworm strains suggests that environmental signals, such as temperature, may affect somatic phenotypes by regulating the hierarchical cascades of Wnt and TGF-β signaling, highlighting the significant role of temperature in individual development and evolution across different animals.

Metabolic rate depression is recognized as a significant physiological mechanism associated with resistance to environmental stress, characterized by a reduction in energy consumption while maintaining essential organismal functions [47,48]. In this study, KEGG enrichment analysis revealed that numerous downregulated Evo_TPG and Evo_non-PG genes were involved in metabolic pathways, particularly carbohydrate metabolism (Table S4). Specifically, downregulated genes from both Evo_TPG and Evo_non-PG were enriched in pathways related to pyruvate metabolism, the citrate cycle (TCA cycle), and glycolysis/gluconeogenesis. Notably, pyruvate is converted to phosphoenolpyruvate, which serves as a precursor for both gluconeogenesis and oxaloacetate formation in the TCA cycle. Therefore, the downregulation of genes in these pathways is likely to reduce glucose production. In addition, pathways such as starch and sucrose metabolism, galactose metabolism, and pentose and glucuronate interconversions were also enriched (Table S3). Furthermore, this metabolic reduction may contribute to decreased cocoon silk synthesis and reproductive performance in resistant silkworms, suggesting that evolutionary changes in metabolic rates may have significant ecological and evolutionary implications.

Energy supply allocation is particularly important for populations that exhibit resistance, as they often endure stressful conditions while maintaining basic development and survival [49]. During the long-term adaptation to high temperature environments, silkworms must overcome oxidative stress and immune challenges posed by pathogenic proliferation. In this study, we hypothesize that resistant silkworms may allocate more resources to survival, which could be a key mechanism of their physiological resistance to high temperatures. Compared to sensitive silkworms, the Evo_non-PG genes with higher basal expression levels in resistant silkworms are primarily enriched in pathways associated with autophagy, mitophagy, apoptosis, ubiquitin-mediated proteolysis, lysine degradation, phagosome function, and Toll and Imd signaling (Table S4). Autophagy, mitophagy, and apoptosis are important for recycling damaged organelles and promoting programmed cell death [50,51]. Ubiquitin-mediated proteolysis is known to play a significant cytoprotective role by degrading damaged proteins. The phagosome and Toll/Imd signaling pathways are essential for immune responses [52,53]. The increased basal expression levels of these genes in resistant strains may enhance their ability to recycle damaged proteins and cells in response to high temperatures while improving immunity. Although maintaining high expression levels in these pathways is energetically costly, it may enable resistant silkworms to respond quickly and effectively to frequent and irregular thermal stimuli. In tropical and subtropical environments, even with metabolic rate depression, resistant silkworms may prioritize resource allocation for survival, addressing protein and cellular damage as well as threats from exogenous pathogens.

Although insects are classified as ectotherms or poikilotherms, they possess physiological mechanisms that enable thermoregulation, such as cuticular transpiration and ventilation through the tracheal system [6]. Generally, evaporative cooling via tracheal ventilation is the primary method of thermoregulation in high temperature environments [6,54]. However, earlier studies have demonstrated that insects can maintain their internal temperature effectively in dry air under high temperatures, while in saturated air, their internal temperature tends to exceed that of the surrounding air, indicating that high humidity impairs evaporative cooling [6,22]. Insect cuticular proteins are major components of the cuticle and play a vital role in protecting internal structures while minimizing water loss through transpiration [55]. In this study, we identified 18 cuticular proteins among the 88 Evo_TPG genes (Table S3), of which 17 were found to be downregulated in resistant silkworms after exposure to high temperatures. The tissue expression patterns revealed that nearly all 18 thermoplastic cuticular protein genes were highly expressed in the head and/or epidermis (Figure S4). For resistant silkworms, long-term adaptation to hot and humid environments may involve a strategy where the downregulation of thermoplastic cuticular proteins reduces cuticle thickness, thereby increasing transpiration when tracheal ventilation is inhibited by high humidity, which may help lower internal temperatures.

Compared to high temperature, high humidity is a relatively weaker environmental factor for silkworms, resulting in fewer DEGs. However, when high temperature and high humidity coexist, even resistant strains exhibit more severe survival effects than when exposed to high temperature alone (Figure S2D). In this study, we investigated the common stress response genes associated with both high temperature and high humidity across the three strains and identified only one gene (Figure S8D). When comparing the two resistant strains, we identified 23 DEGs, with 16 of these exhibiting consistent expression patterns (Figure S8E). Several notable genes were annotated, including *SOH1* and *Sds3*. *SOH1* has been reported to be involved in repressing the temperature-dependent growth of the HPR1 mutant in *Saccharomyces cerevisiae* [56] and is also implicated in DNA repair [57]. *Sds3* functions as a component of the co-repressor complex of histone deacetylases and can repress gene transcription. We examined the KEGG enrichment of stress response genes related to high temperature and high humidity in the resistant strains 932 and 7532 and found that almost all genes associated with metabolic pathways were downregulated, including those involved in the TCA cycle and carbon metabolism (Table S2 and Table S7). Interestingly, both *SOH1* and *Sds3* were upregulated under conditions of high temperature and high humidity in the resistant silkworms (Table S3), suggesting that these genes may play a significant role in reducing developmental energy expenditure to cope with the combined stresses of elevated temperature and humidity.

Generally, genetic diversity in genomic sequences decreases in a population after natural or artificial selection. However, genetic diversity in selected regions tends to increase significantly when comparing populations that have undergone selection to those that have not [28,58]. In this study, the two resistant strains, 7532 and 932, experienced similar selection pressures in high temperature environments. At the basal expression level, 1338 genes exhibited differential expression between the resistant and sensitive strains (Figure 7B). As a result of natural selection, nucleotide divergence in the Evo_non-PG gene regions and their upstream sequences led to a significant increase between the resistant and sensitive strains (Figure 10C,D), consistent with the genetic variation observed in selected genes in previous studies [28,58]. In contrast, the upstream regions of the Evo_TPG exhibited comparable nucleotide divergence (Figure 10B). Theoretically, the upstream regions of these genes may experience positive selection, which could result in significant differences between the resistant and sensitive strains. However, our observations revealed only a slight increase in nucleotide divergence. For instance, the average substitution rate in the upstream regions between strains 7532 and HY was 17.95 bp/Kb, while the rate for randomly selected genes was 15.26 bp/kb (ANOVA, *p* = 0.08), indicating that variations in transcription patterns may arise from minor sequence changes in the upstream regulatory regions, or the specific regulation of Evo_TPG genes in resistant strains may be influenced by changes in the expression of trans-regulatory factors. Collectively, these results suggest that the genes that evolved in the resistant strains and adapted to thermal environments, may have undergone strong natural selection, leading to increased nucleotide divergence between the resistant and sensitive strains, which may facilitate adaptations to high temperatures, similar to findings in Drosophila [15]. Future sequencing of additional resistant and sensitive strains could enable a comprehensive analysis of positive selection signals across the genome, thereby deepening our understanding of the underlying evolutionary mechanisms.

## 5. Conclusions

In this study, the silkworm was used as a model species to investigate its evolutionary response and molecular mechanisms of adaptation to humid tropical/subtropical climates. The findings revealed that resistant silkworms from tropical/subtropical humid regions primarily exhibited higher tolerance to high temperatures, with higher CTmax values, and tended to have lower larval weight, size, cocoon silk yield, and spawning capacity compared to the sensitive strain. Transcriptome analysis revealed that 88 Evo_TPGs and 1338 Evo_non-PGs contributed to the development of heat tolerance in the silkworms. Functional annotations of the evolved genes indicated that resistant silkworms could improve their physiological tolerance to heat primarily through mechanisms such as suppressing metabolic rates, reducing developmental energy allocation, and increasing cuticle transpiration to dissipate internal heat, but at the expense of other survival traits such as larval size and cocoon yield. In addition, the resistant silkworms could allocate more of their limited resources to counteract high temperature-induced oxidative stress damage, allowing them to respond quickly and efficiently to frequent and irregular thermal stimuli. Furthermore, the evolved genes showed significantly higher nucleotide divergence between resistant and sensitive strains compared to randomly selected genes, suggesting that these genes might have undergone selection during evolution. Moreover, only 10 and 16 differentially expressed genes in the two resistant strains showed consistent stress responses under conditions of high humidity and the interaction of high temperature and high humidity. Among them, *SOH1* and *Sds3* were found to play an important role in reducing developmental energy expenditure to cope with severe temperature and humidity stress. In summary, these findings provide important insights into understanding the adaptive evolution of insects in response to climate change and could help improve insect survival predictions under the ongoing threat of global warming.

## Data Availability

The RNA-seq data obtained in the present study were deposited in the NCBI Sequence Read Archive (SRA, https://www.ncbi.nlm.nih.gov/sra/, accessed on 2 October 2024.) under the accession number PRJNA1167689, and the genome re-sequencing datasets were accessed by PRJNA1167798.

## Supplementary Materials

The following supporting information can be downloaded at: https://www.mdpi.com/article/10.3390/insects16090962/s1. Figure S1: Survival probability between resistant strain 932 and sensitive strain Haoyue under high temperature and humidity treatment. (A) Summary of all the survival curves. The *x*-axis represents treatment days for the fifth instar larvae. (B) Differences in survival probability between the two strains. The *p*-values were computed by the log-rank test. (C) Survival probability between males and females. (D) Survival probability under two treated conditions. Figure S2: Survival probability between two tolerant strains, 7532 and 932, under high temperature and humidity treatment. (A) Summary of all the survival curves. The *x*-axis represents treatment days for the fifth instar larvae. (B) Differences in survival probability between the two strains. The *p*-values were computed by the log-rank test. (C) Survival probability between males and females. (D) Survival probability under two treated conditions. Figure S3: Spawning number and hatchability of the three silkworm strains after medium-high temperature and high humidity. (A) Effect of the medium-high temperature and high humidity on spawning number. The fifth-instar individuals were reared under 25 °C/70% RH and 30 °C/90% RH conditions, respectively. (B) Comparison of hatchability. * *p* < 0.05; ns: not significant. Figure S4: Expression pattern of the cuticular proteins in the 5L3D larvae. The upregulated genes induced by heat stress were shown in red, and the downregulated genes were shown in black. Figure S5: Expression heatmap and functional enrichment of the thermoplastic genes in both the resistant and sensitive strains. A total of 146 TPG_RS genes were identified, which showed the same stress response in resistant and sensitive strains. (B) Significantly enriched KEGG pathways and GO terms of the TPG_RS genes were displayed. BP: biological process, CC: cellular component, MF: molecular function. Figure S6: Soft clusters of all the DEGs respond to humid stress by Mfuzz software. Red and purple lines correspond to genes with high membership value. The numbers on the *x*-axis represent the treated humidities, including 70% RH, 80% RH, and 90% RH. All the treatments were kept under 25 °C. Figure S7: The evolved humid-plastic genes and expression patterns. (A) DEG number comparisons in 932, 7532, and Haoyue after different humidity exposures. (B) Expression patterns of the six overlapping DEGs among the three strains after different humidity exposures. (C) Expression patterns of the 10 evolved humid-plastic genes. In total, 36 overlapping genes were found between the two resistant strains in (A), while only 10 genes exhibited a consistent humid-plastic response, which might be the evolved humid-plastic genes in the resistant silkworms. (D) Expression patterns of the evolved humid-plastic genes in the various tissues of the fifth-instar larvae. Figure S8: Specific response genes of the resistant strains after high temperature and high humidity exposure. (A−C) DEG number comparisons in 932, 7532, and Haoyue after high temperature and/or high humidity exposure. The green parts represent the number of specific response genes under 35 °C/90% RH in each strain. In total, 1168, 425, and 576 specific response genes were found in 932, 7532, and Haoyue, respectively. (D) Venn diagram of the specific response genes of the three strains. In total, 23 overlapping genes between two resistant strains after exposure to high temperature and high humidity, of which 16 genes exhibited consistent expression patterns (E). Figure S9: Comparison of individual weight, cocoon yield, and spawning number among the three silkworm strains under normal rearing conditions. Comparison of larval individual weight of females (A) and males (B). Comparison of pupal individual weight of females (A) and males (B). Whole cocoon weight (WCW) of females (E) and males (F). Cocoon shell weight (CSW) of females (G) and males (H). (I) Comparison of spawning number among the three silkworm strains. ** *p* < 0.01; * *p* < 0.05; ns: not significant. Table S1: Accession numbers of the known transcriptome dataset in the various tissues on day 3 of the fifth instar larvae. Table S2: ALL the DEGs in the three silkworm strains after different temperature and humidity exposure. Table S3: Functional annotations of the evolved and plastic response genes. “Response to hThH in R” represents specific response genes of the resistant strains after high temperature and high humidity exposure. Table S4: KEGG enrichment of the evolved and plastic response genes. Table S5: GO enrichment of the evolved and plastic response genes. Table S6: Putative transcription factor (TF) within the evolved and plastic response genes. Potential TFs were predicted using the AnimalTFDB database (http://bioinfo.life.hust.edu.cn/AnimalTFDB/). Table S7: KEGG enrichment of the specific response genes in each strain after high temperature and high humidity exposure. Table S8: Functional information and enriched tissues of the evolved and plastic response genes. If a gene belonged to an enriched pathway in Table S4, the pathway name was shown in red.
