# Supplementary material for: Adaptation to Hot and Humid Climates in the Silkworm: Energy Reallocation and Cuticle Transpiration
Source: Insects. 2025 Sep 12;16(9):962. doi: 10.3390/insects16090962 (PMC12470560; doi:10.3390/insects16090962)
Supplement: Supplementary file 1 [file insects-16-00962-s001.zip › Figure S6.pdf]

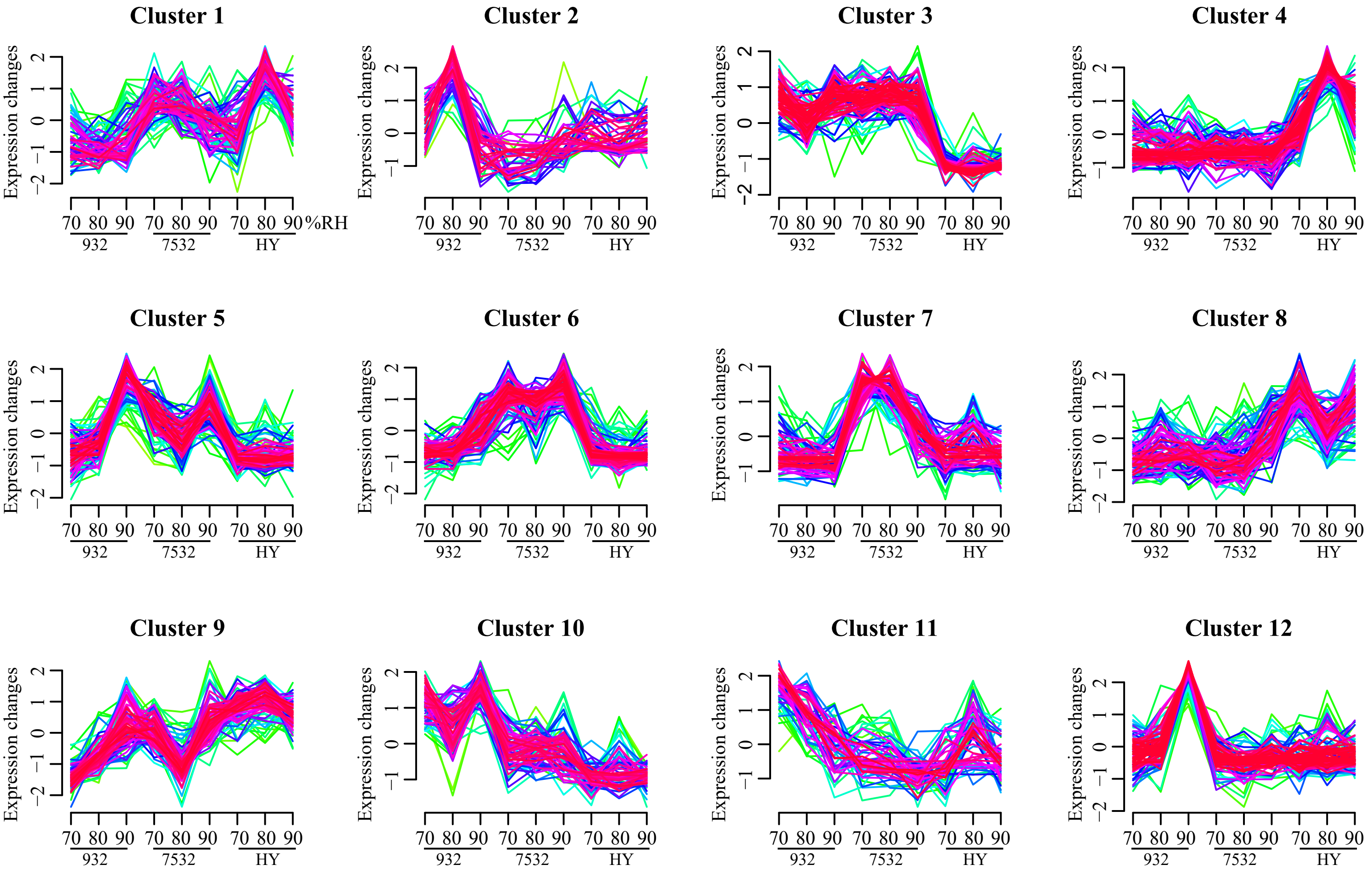
Cluster 1
Cluster 2
Cluster 3
Cluster 4
Cluster 5
Cluster 6
Cluster 7
Cluster 8
Cluster 9
Cluster 10
Cluster 11
Cluster 12
Expression changes
70 80 90 70 80 90 70 80 90 %RH
932
7532
HY
