# Supplementary material for: Adaptation to Hot and Humid Climates in the Silkworm: Energy Reallocation and Cuticle Transpiration
Source: Insects. 2025 Sep 12;16(9):962. doi: 10.3390/insects16090962 (PMC12470560; doi:10.3390/insects16090962)
Supplement: Supplementary file 1 [file insects-16-00962-s001.zip › Figure S9.pdf]

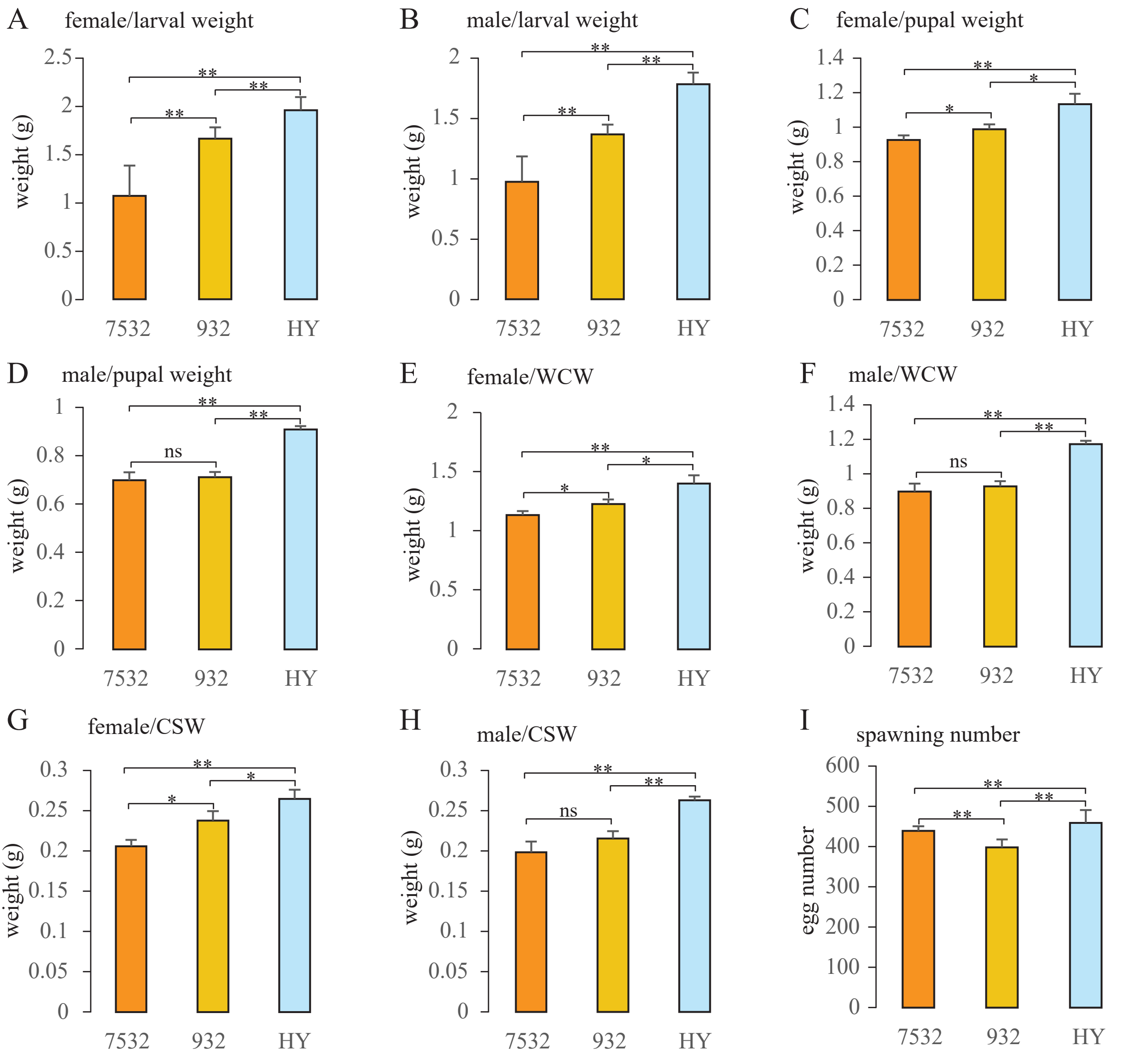
A
female/larval weight
weight (g)
B
male/larval weight
C
female/pupal weight
D
male/pupal weight
E
female/WCW
F
male/WCW
G
female/CSW
H
male/CSW
I
spawning number
egg number
7532
932
HY
ns
