# Supplementary figures and images for: Adaptation to Hot and Humid Climates in the Silkworm: Energy Reallocation and Cuticle Transpiration
Source: Insects. 2025 Sep 12;16(9):962. doi: 10.3390/insects16090962 (PMC12470560; doi:10.3390/insects16090962)

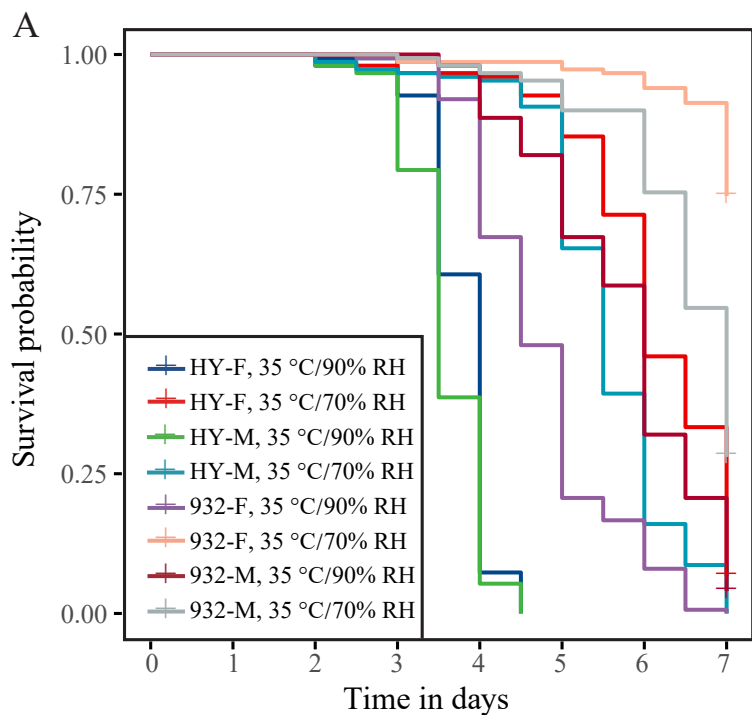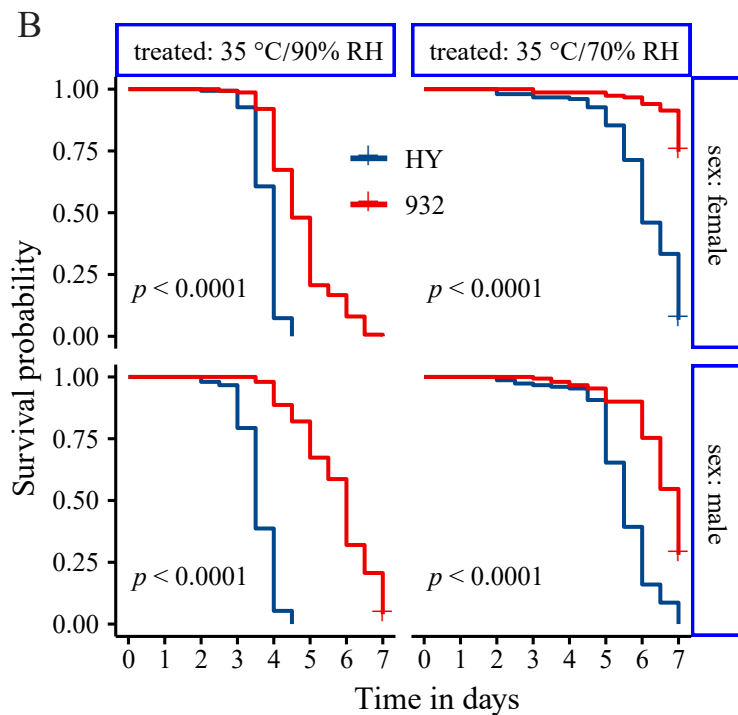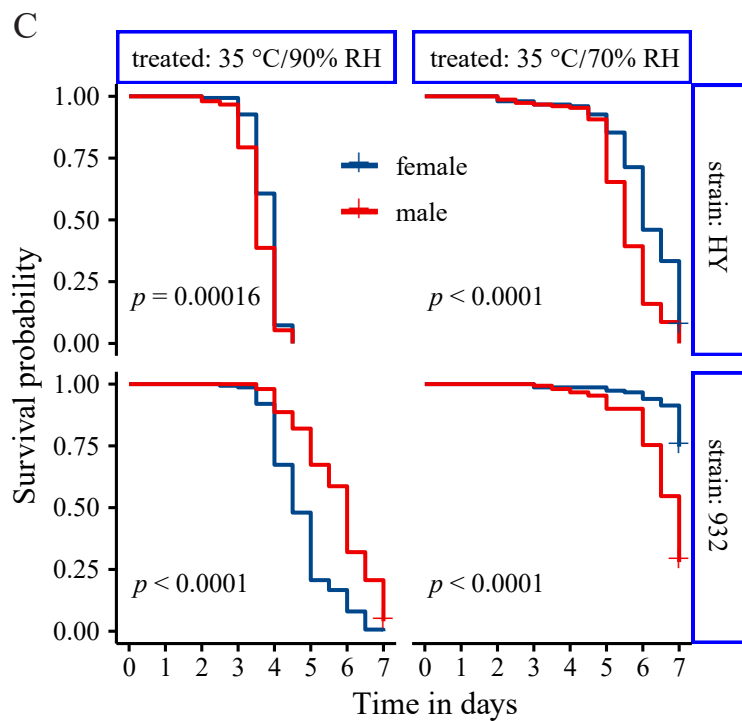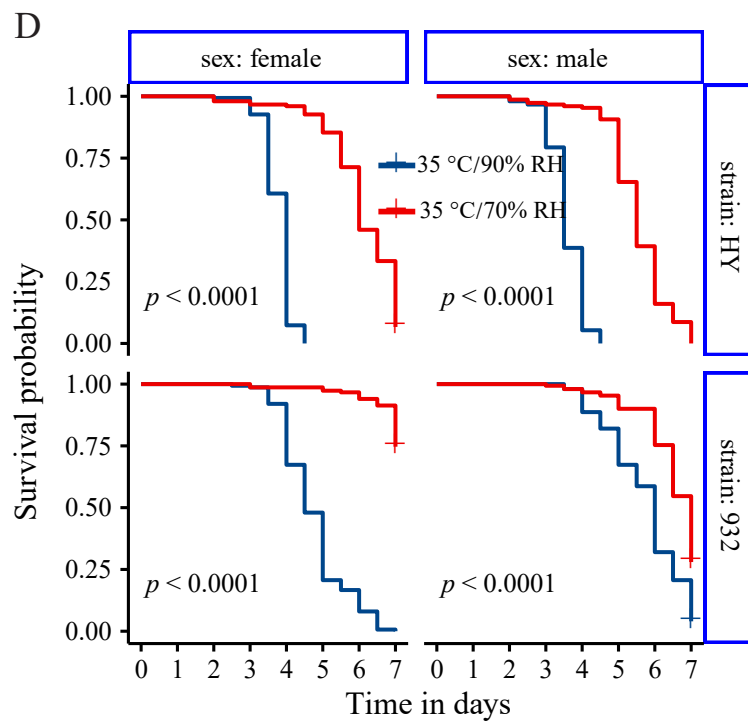

Supplement: Supplementary file 1 [file insects-16-00962-s001.zip › Figure S1.pdf]

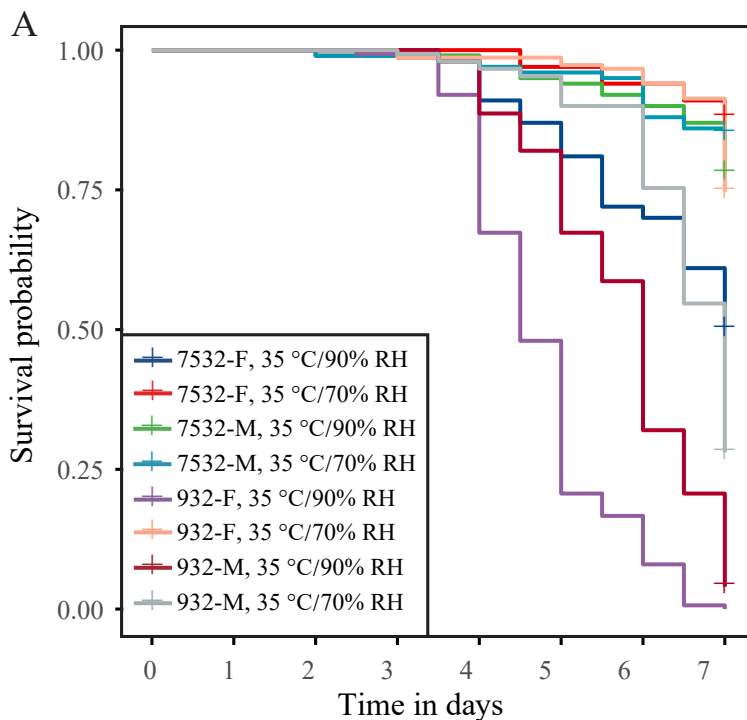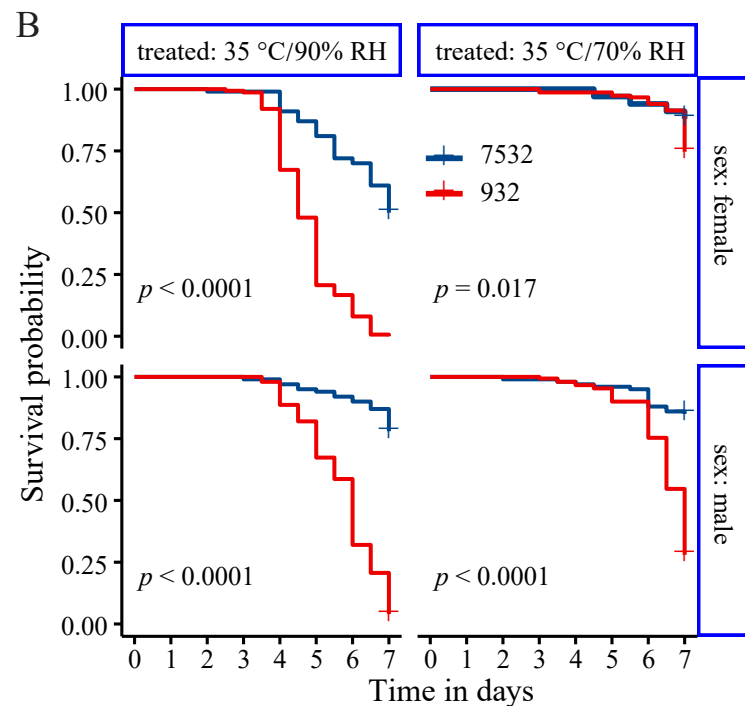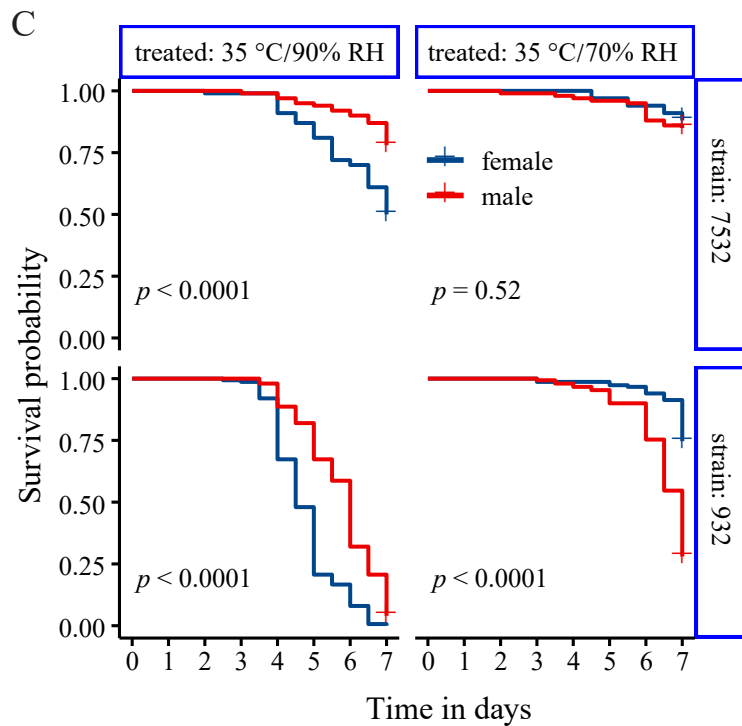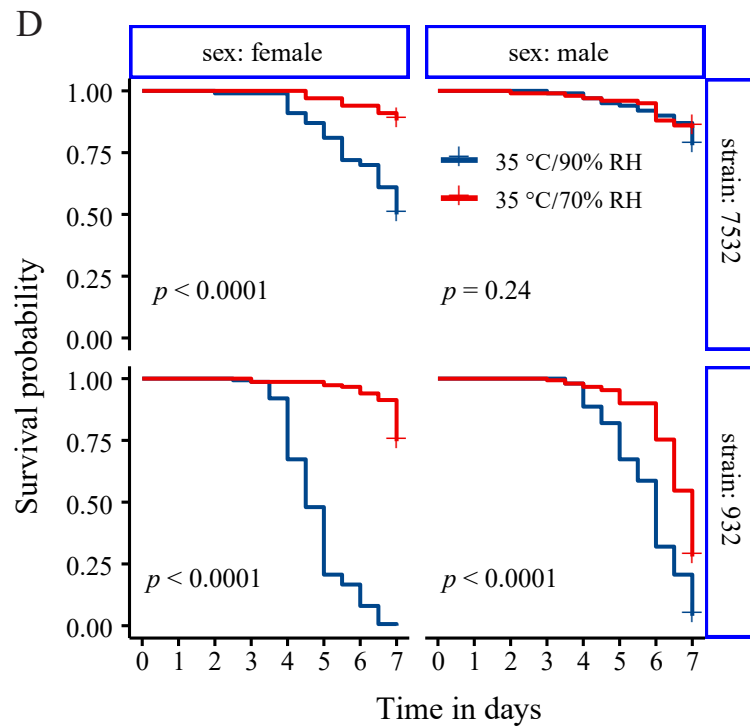

Supplement: Supplementary file 1 [file insects-16-00962-s001.zip › Figure S2.pdf]

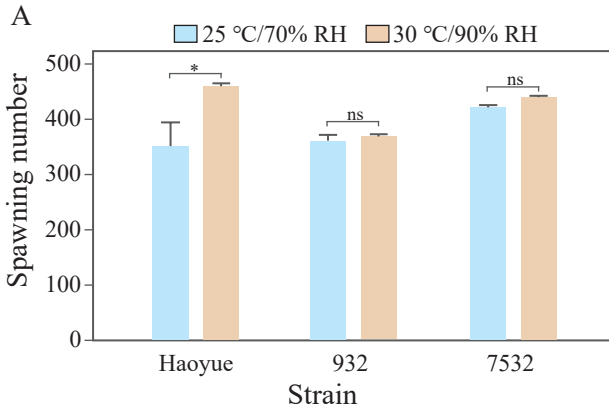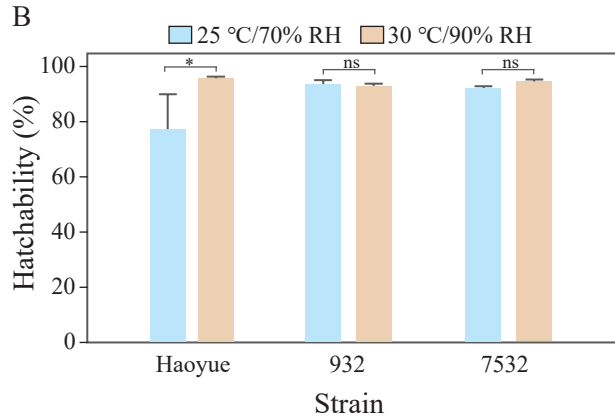

Supplement: Supplementary file 1 [file insects-16-00962-s001.zip › Figure S3.pdf]

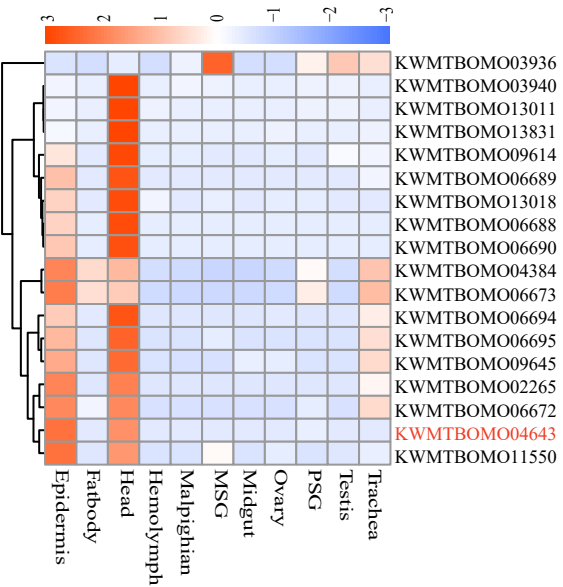

Supplement: Supplementary file 1 [file insects-16-00962-s001.zip › Figure S4.pdf]

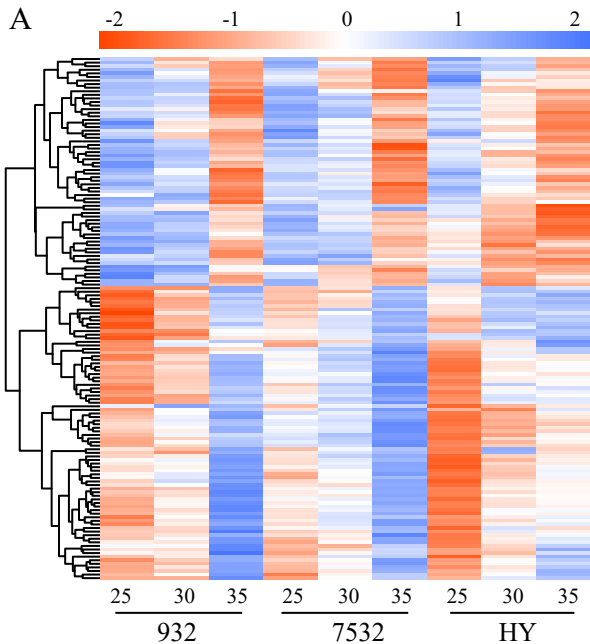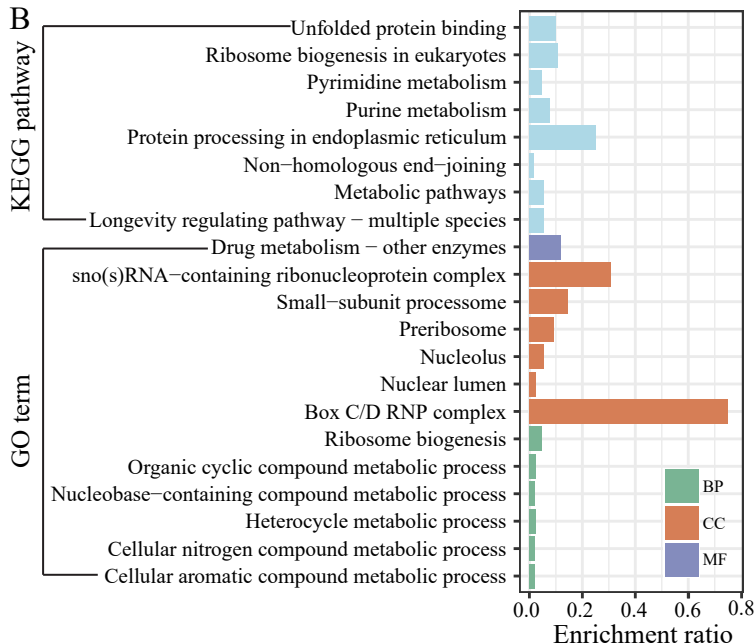

Supplement: Supplementary file 1 [file insects-16-00962-s001.zip › Figure S5.pdf]

A

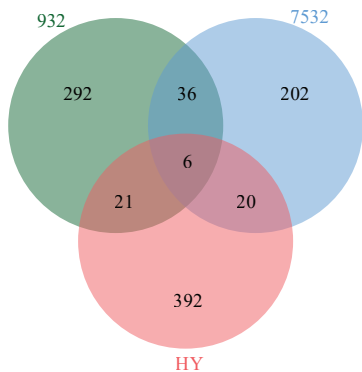

B

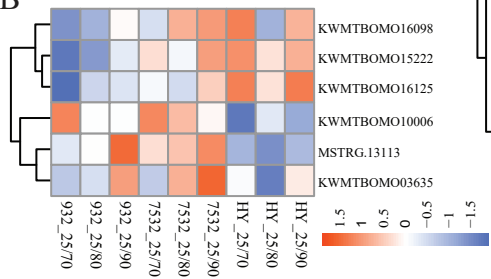

C

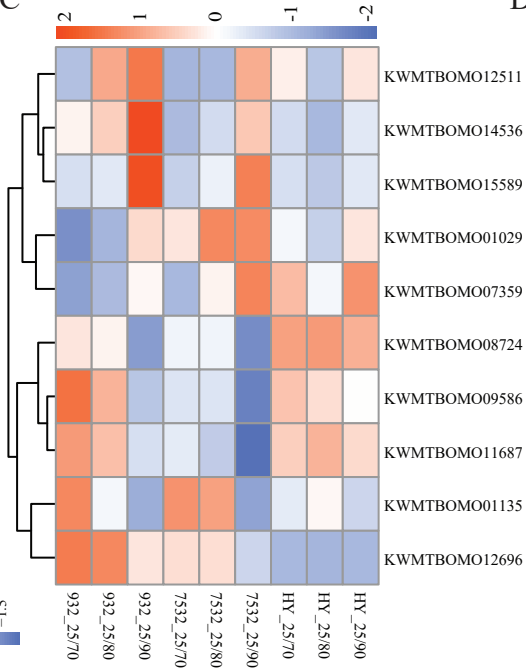

D

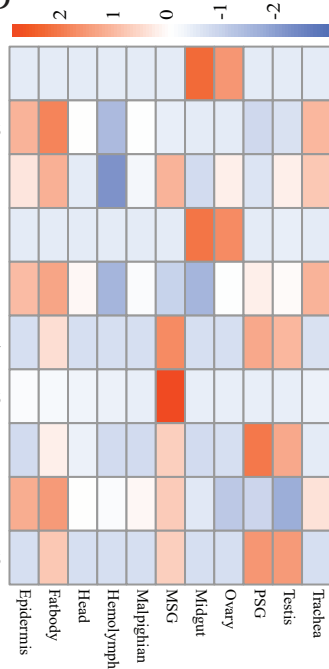

Supplement: Supplementary file 1 [file insects-16-00962-s001.zip › Figure S7.pdf]

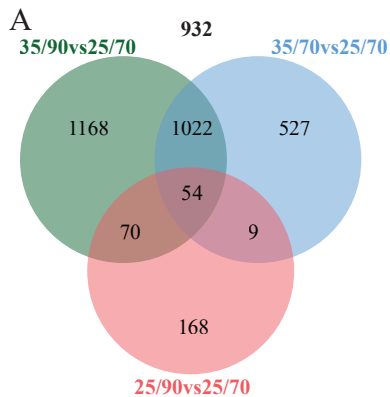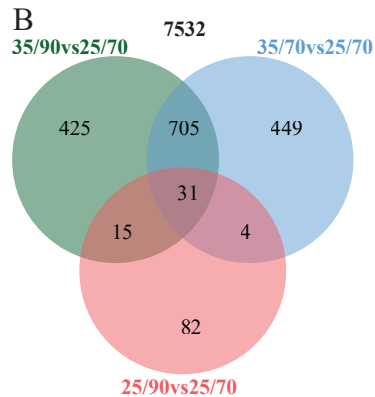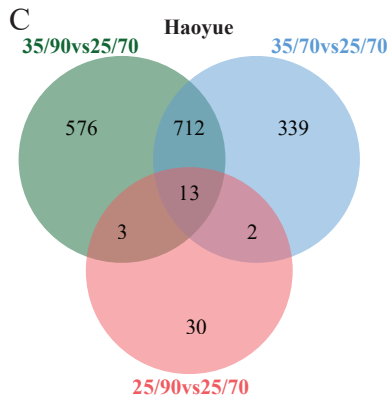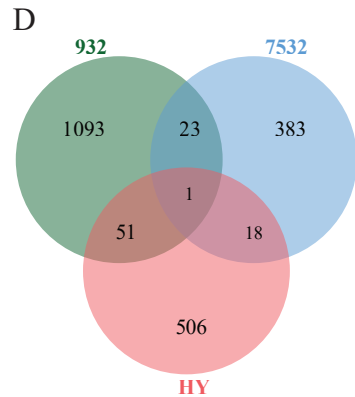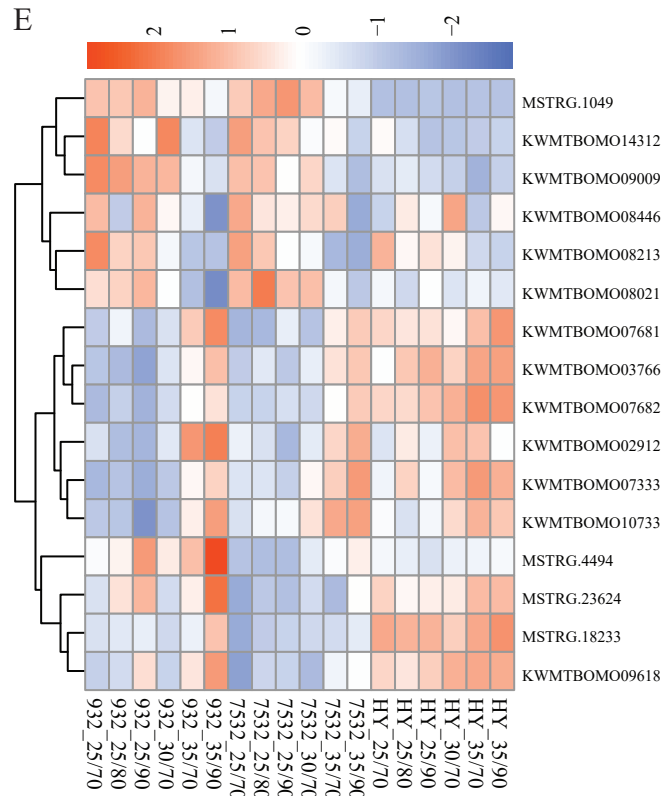

Supplement: Supplementary file 1 [file insects-16-00962-s001.zip › Figure S8.pdf]
